# A polyphenol-rich plant extract prevents hypercholesterolemia and modulates gut microbiota in western diet-fed mice

**DOI:** 10.3389/fcvm.2024.1342388

**Published:** 2024-01-22

**Authors:** Cédric Langhi, Marie Vallier, Auriane Bron, Yolanda F. Otero, Maheva Maura, Florian Le Joubioux, Niek Blomberg, Martin Giera, Bruno Guigas, Thierry Maugard, Benoit Chassaing, Sébastien Peltier, Stéphanie Blanquet-Diot, Jean-Marie Bard, Pascal Sirvent

**Affiliations:** ^1^ R&D Riom Center, Valbiotis, Riom, France; ^2^ UMR 454 Microbiologie Environnement DIgestif et Santé (MEDIS), Université Clermont Auvergne, Clermont-Ferrand, France; ^3^ R&D Center, Valbiotis, La Rochelle, France; ^4^ Center for Proteomics and Metabolomics, Leiden University Medical Center, Leiden, Netherlands; ^5^ Department of Parasitology, Leiden University Medical Center, Leiden, Netherlands; ^6^ Equipe BCBS (Biotechnologies et Chimie des Bioressources Pour la Santé), UMR CNRS 7266 LIENSs, La Rochelle Université, La Rochelle, France; ^7^ Team “Mucosal Microbiota in Chronic Inflammatory Diseases”, Institut Cochin, INSERM U1016, CNRS UMR 8104, Université Paris Cité, Paris, France; ^8^ R&D Périgny Center, Valbiotis, Périgny, France; ^9^ Laboratoire de Biochimie Générale et Appliquée, UFR de Pharmacie, ISOMer-UE 2160, IUML-Institut Universitaire Mer et Littoral-FR3473 CNRS, Université de Nantes, Nantes, France

**Keywords:** cholesterol, triglycerides, lipoproteins, intestine, microbiota

## Abstract

**Introduction:**

Totum-070 is a combination of five plant extracts enriched in polyphenols to target hypercholesterolemia, one of the main risk factors for cardiovascular diseases. The aim of this study was to investigate the effects of Totum-070 on cholesterol levels in an animal model of diet-induced hypercholesterolemia.

**Methods:**

C57BL/6JOlaHsd male mice were fed a Western diet and received Totum-070, or not, by daily gavage (1g/kg and 3g/kg body weight) for 6 weeks.

**Results:**

The Western diet induced obesity, fat accumulation, hepatic steatosis and increased plasma cholesterol compared with the control group. All these metabolic perturbations were alleviated by Totum-070 supplementation in a dose-dependent manner. Lipid excretion in feces was higher in mice supplemented with Totum-070, suggesting inhibition of intestinal lipid absorption. Totum-070 also increased the fecal concentration of short chain fatty acids, demonstrating a direct effect on intestinal microbiota.

**Discussion:**

The characterization of fecal microbiota by 16S amplicon sequencing showed that Totum-070 supplementation modulated the dysbiosis associated with metabolic disorders. Specifically, Totum-070 increased the relative abundance of *Muribaculum* (a beneficial bacterium) and reduced that of *Lactococcus* (a genus positively correlated with increased plasma cholesterol level). Together, these findings indicate that the cholesterol-lowering effect of Totum-070 bioactive molecules could be mediated through multiple actions on the intestine and gut microbiota.

## 1 Introduction

Hypercholesterolemia is strongly associated with atherosclerosis. This disease leads to myocardial infarction, stroke and peripheral vascular disease. In people with markedly elevated cholesterol levels, treatment with cholesterol-lowering medication is usually prescribed. For individuals with moderately or slightly elevated cholesterol levels, lifestyle interventions, such as physical activity, dietary changes and dietary supplements, are recommended (1). Plant-based supplements are tested as a nutritional solution for disease prevention. Indeed, phytochemicals that lower serum glucose, cholesterol, and triglyceride concentrations are considered to reduce the risk of cardiovascular diseases (2). Many plants have been investigated to reduce blood cholesterol (3–6). Artichoke (*Cynara scolymus L*) is a plant rich in natural antioxidants. The pharmacological properties of artichoke leaf extracts are well documented and might contribute to lower cholesterol levels in humans (7, 8). The beneficial effects of artichoke leaf extract are largely explained by their polyphenolic compounds, namely, chlorogenic acid, caffeic acid, 3,4-di-*O*-caffeoylquinic acid and cynarine, and the main flavonoids apigenin and luteolin (9, 10). Moreover, several studies suggest that olive leaf extracts can reduce cardiovascular disease risk factors, notably through their hypocholesterolemic effect (11, 12). The main phenolic compounds present in olive plant (*Olea europaea L*) are luteolin, oleuropein, tyrosol, and hydroxytyrosol. Olive leaves contain the highest concentrations of these compounds, especially oleuropein, compared with other parts of the plant (13). Goji berries (*Lycium barbarum*) have been traditionally used as food and medicinal plants in China and other Asian countries (14). The various pharmacological actions of these berries are mainly attributed to the presence of polysaccharides, flavonoids and carotenoids, especially zeaxanthin, quercetin, and rutin (15, 16). Goji berries also contain betaine, an alkaloid that helps to produce choline, a compound that strengthens the liver. Goji berries significantly reduce total cholesterol and total triglyceride concentrations in older adults (17). Black pepper (*Piper nigrum L*.) is commonly used as a spice in various food types. The main bioactive ingredient of black pepper fruits (i.e., piperine) has a wide range of pharmacological effects, including anti-oxidant and cholesterol-lowering properties (18). Piperine can enhance the absorption of vitamins and polyphenols (19). Chrysanthellum (*Chrysanthellum indicum subsp. afroamericanum B.L. Turner*) is a plant that is widespread in the tropics and that reduces cholelithiasis and acts as a hypolipidemic agent (20).

Totum-070 is a polyphenol-rich blend of these five plant extracts (olive leaves, artichoke leaves, chrysanthellum, goji fruits and black pepper) selected because of their abundance in bioactive molecules with the aim of lowering cholesterol in individuals with mild to moderate hypercholesterolemia (French Priority Patent Number: FR1559965). Recently, Wauquier et al. demonstrated that administration of 3 g/kg Totum-070 prevents the development of dyslipidemia in hamsters fed a high-fat diet (HFD) for 12 weeks (21). Moreover, using an explorative clinical approach in healthy volunteers who received a single dose of Totum-070 (5 g), the authors detected the presence of about twenty circulating metabolites, including chlorogenic acid, cynarine, luteolin glucuronides isomers, oleuropein glucuronides isomers, hydroxytyrosol sulfate isomers, and hydroxytyrosol glucuronides isomers. In addition, the *ex vivo* incubation of palmitate-exposed hepatocytes with human sera enriched in these bioactive molecules following Totum-070 ingestion limited the accumulation of intracellular lipids (21).

Plasma cholesterol concentration is influenced by several factors, including the absorption of dietary cholesterol, endogenous cholesterol synthesis, cholesterol removal from the circulation, and the conversion of hepatic cholesterol into bile acids followed by excretion in the feces (22). Accumulating data from human and animal studies have demonstrate that intestinal microbes can affect the host cholesterol metabolism through multiple direct and indirect biological mechanisms (23, 24). Interestingly, many plant extracts can mitigate dyslipidemia and obesity by regulating the gut microbiota relative abundance and composition (25). There is evidence from animal studies that certain doses of selected polyphenols and polyphenol-rich plant extracts modify the gut microbial composition. For example, supplementation with water extract of Goji fruits ameliorated weight gain and insulin resistance in HFD-fed mice (26). Goji extracts elevated hepatic bile acid biosynthesis and excretion of bile acids in association with decreased relative abundance of *Clostridium_XIVa*. In rabbits, Goji berry supplementation modulates gastrointestinal microbiota composition and cecal fermentation, with the families Ruminococcaceae and Lachnospiraceae relatively more abundant with Goji than in control group (27). In HFD-fed mice, administration of Olive leaf extract was able to counteract the altered composition in the gut microbiota associated to obesity and restored the main bacteria phyla to the normal values observed in standard diet-fed mice (28). Supplementation with chlorogenic acid in HFD-fed mice protected against metabolic disorders and endotoxemia. Chlorogenic acid significantly changed the composition of the gut microbiota and increased the relative abundance of short chain fatty acid (SFCA)-producers (29). In another study in mice, chlorogenic acid also reversed the HFD-induced gut microbiota dysbiosis, including significantly inhibiting the growth of Desulfovibrionaceae, Ruminococcaceae, Lachnospiraceae, Erysipelotrichaceae, and raising the growth of Bacteroidaceae and Lactobacillaceae (30).

The aim of the present study was to investigate the effects of Totum-070 supplementation in a murine model of hypercholesterolemia and to explore hypotheses of Totum-070 mechanism of action.

## 2 Materials and methods

### 2.1 Totum-070 (lipidrive ®) characterization

The batch used in this study was chemically characterized using the Folin–Ciocalteu method (total polyphenol estimation), the Dubois method (total sugar estimation), the Phospho-Vanillin method (total lipid estimation) (31, 32), the O-phthalaldehyde method (total protein estimation) (33), and HPLC-UV/visible/MS (Agilent Technologies Infinity series 1,200 and 1,260, Santa Clara, CA, USA) with a C18 column (250 × 4.6 mm, 5 μm, Phenomenex, USA) and a HILIC Silica column (150 × 4.6 mm, 5 μm, Waters, The Netherlands) (quantification of potential compounds of interest). The results of this analysis are presented in Table 1.

**Table 1:** Chemical characterization of Totum-070.

Compound type (sorted by families)	Extract content (g/100 g dry weight)
Total sugars	41.35
Total lipids	7.30
Total Proteins	2.05
Betaine	0.23
Total phenolic compounds	15.42
Monocaffeoylquinic acids	
Chlorogenic acid	0.81
Cryptochlorogenic acid	0.01
Other monocaffeoylquinic acids	0.25
Dicaffeoylquinic acids	
Cynarin	0.16
3–5 dicaffeoylquinic acid	0.18
4–5 dicaffeoylquinic acid	0.14
Other dicaffeoylquinic acids	0.11
Caffeic acid	0.01
Oleuropein	4.6
Oleuropein isomers	0.47
Hydroxytyrosol	0.08
Luteolin	0.01
Luteolin-7-O-glucoside	0.27
Luteolin-7-O-glucoside isomer	0.02
Luteolin-4-O-glucoside	0.06
Luteolin-7-O-glucuronide	0.29
Apigenin-7-O-glucoside	0.03
Apigenin-7-O-glucuronide	0.17
Apigenin-6-C-glucoside-8-C-arabinoside (Shaftoside)	0.01
Apigenin-6,8-C-diglucoside (Vicenin 2)	0.02
Apigenin-7-O-rutinoside	0.01
Eriodictyol-7-O-glucoside	0.04
Flavanomarein	0.14
Marein	0.05
Maritimein	0.05
Rutin	0.02
Verbascoside	0.08
Terpenes and terpenoids	
Oleanolic acid	1.64
Saponins	
Chrysanthellin A	0.28
Chrysanthellin B	0.27
Iridoids	
Oleoside	0.01
Cynaropicrin	0.07
Alkaloids	
Piperine	0.06

### 2.2 Animals

All animal procedures were approved by the local ethics committee (C2E2A, Auvergne, France) and comply with the ARRIVE guidelines. Male C57BL/6JOlaHsd mice were purchased from Envigo (Gannat, France). All mice were housed at Valbiotis animal facility (Valbiotis R&D Center, Riom, France) at 22°C under standard 12 h light-12 h dark cycle. Upon arrival, 56 mice were fed a normal diet (ND) (D14042701, Research Diets, New Brunswick, NJ, USA) for 2 weeks of acclimatization until the age of 8 weeks. Then, animals were randomly assigned to four groups (*n* = 14 mice per group) with similar average body weight and fat mass: (i) ND, (ii) WD (41 Kcal % fat, 30 Kcal % sucrose and 0.21% w/w cholesterol; D12079B, Research Diets, New Brunswick, NJ, USA), (iii) WD supplemented with 1 g/kg of Totum-070 (WD-T070 1 g/kg), and (iv) WD supplemented with 3 g/kg of Totum-070 (WD-T070 3 g/kg). Totum-070 solution was prepared in vehicle (1% Tween-20 in tap water) at 100 g/L or 300 g/L. Totum-070 was administered to WD-fed mice by gavage daily. Control groups (WD and ND) received only vehicle by gavage. Food and water were supplied *ad libitum*. Supplementation was for 6 weeks. Body weight was recorded weekly, and food intake was measured 3 times per week. The ND and WD energy densities were 3.9 kcal·g^−1^ and 4.7 kcal·g^−1^, respectively. Plasma lipid parameters were monitored before the study start (week 0), after 3 weeks of supplementation (week 3), and at the study end (week 6). Mice were fasted for 6 h and blood was collected from the tail vein into EDTA-treated capillaries. Blood was centrifuged at 2,000 g at 4°C for 10 min. Plasma was harvested and stored at −80°C until analysis. Body composition was assessed with an echo MRI system (Zynsser Analytic, Frankfurt, Germany). At the end of the 6 weeks of supplementation, animals were fasted for 6 h, anaesthetized with isoflurane, and euthanized by cervical dislocation.

### 2.3 Plasma lipid parameters

Cholesterol levels in the plasma were quantified enzymatically using the CHOD-PAP colorimetric assay kit (Biolabo SAS, Maizy, France). Plasma triglyceride concentration was measured using a colorimetric assay kit (Cayman chemical, Ann Arbor, MI, USA).

### 2.4 Liver triglycerides

Liver triglyceride level was determined with the triglyceride assay kit by Cayman Chemical. Mouse livers (100 mg) were homogenized in 1 ml of a solution containing 5% NP40 in water followed by centrifugation (10,000 g at 4°C for 10 min) to recover the supernatant. Triglyceride concentration was normalized to the liver sample weight and expressed as mg/g liver weight.

### 2.5 Histology

At the study end, liver tissue was fixed in paraformaldehyde at 4°C for 48 h and kept in 70% ethanol for 2 to 3 h before automated tissue processing. Samples were embedded in paraffin and 4*μ*m sections were cut with a microtome. Sections were dewaxed and rehydrated in successive xylene and ethanol baths. Then, they were stained in Gill II hematoxylin for 4 min, and after removal of excess stain in an alcohol acid bath, they were transferred to a 0.25% eosin Y bath for 45 s. Finally, sections were dehydrated with ethanol and xylene, and slides were mounted with the Eukitt medium. Other liver tissue samples were frozen in OCT medium, and 10 μm tissue sections were cut with a cryostat and fixed in cold 10% formalin for 5 min. Slides were stained with Oil Red O at 60°C in an oven for 8 min. Oil Red O areas were quantified using the ImageJ software. Stained areas of at least 10 μm² and with a circularity between 0.1 and 1 were selected for quantifying the Oil Red O surface as percentage of the total surface. At least three images per liver were analyzed.

### 2.6 Quantitative reverse transcription PCR (Rt-qPCR)

Total mRNA was extracted from snap-frozen tissues using TRIzol® (Invitrogen, Life Technologies). cDNA was synthesized from 2 μg RNA with the High-Capacity cDNA transcription kit (Applied Biosystems, Life Technologies). PCR amplification was carried out using the CFX system (Bio-Rad, Marnes-la-Coquette, France) with Taqman probes (Applied Biosystems, Life Technologies, Carlsbad, CA, USA) and the ΔΔCt method was used to quantify mRNA levels. Gene expression was normalized to the housekeeping genes *Gapdh* (liver samples) and *Polr2a* (intestine samples).

### 2.7 Protein extraction and western blot analysis

Liver samples were homogenized in 20 µl/mg RIPA buffer (50 mM Tris-HCl, 150 mM NaCl, 1% NP40, 0.5% sodium deoxycholate, 0.1% SDS) on ice using a glass potter. Just before use, protease (P8340, Sigma, Saint-Louis, MO, USA) and phosphatase (88,667, Thermo Fisher Scientific, Whaltham, MA, USA) inhibitors were added to the buffer. Homogenized samples were centrifuged at 14,000 g at 4°C for 10 min, and supernatant was collected. Protein content was determined using a commercial Lowry-based DC protein assay (Bio-Rad, Marnes-la-Coquette, France). All samples were adjusted to a standard concentration (15 µg) and then diluted with 2× Laemmli buffer. Western blotting was performed as described by Chavanelle et al. (34) with the following specificities: membranes were incubated overnight at 4°C with primary antibodies against LDL Receptor (Biovision, Milpitas, CA, USA) diluted to 1:1,000. After overnight incubation, membranes were washed with Tris-buffered saline/0.5% Tween-20 and incubated with anti-rabbit horseradish peroxidase-conjugated secondary antibodies (1:2,000) at room temperature for 1 h. Then, membranes were washed three times in Tris-buffered saline/0.5%Tween-20 before addition of an enhanced chemiluminescent solution (Clarity Western ECL; Bio-Rad) for 1 min. Images were acquired with the Bio-Rad ChemiDoc system, and band density was determined using Image Lab V6.0 (Bio-Rad). Stain-free blot images were used as total protein loading control for data normalization (35).

### 2.8 Fecal lipid content

For each cage (1 animal per cage), feces were collected over a week at the study end. Feces were dried at 60°C for 72 h. Fecal lipid quantification was performed using 0.5 g of dried sample per cage by chloroform/methanol lipid extraction as described previously (36). The lipid phase was placed in a glass recipient and solvent was evaporated. The total lipid mass per g of feces was calculated by subtracting the empty glass recipient weight.

### 2.9 Quantification of fecal short chain fatty acids (SCFAs)

Lyophilized feces (± 50 mg) were mixed in 750 µl of 0.04 M sulfuric acid using an Ultra-Turrax® (Ika, Staufen, Germany). Samples were centrifuged (16,000 g, 4°C, 15 min) and the supernatants were filtered (0.22 μm). The three major SCFAs (acetate, propionate and butyrate) were quantified by high-performance liquid chromatography (HPLC, Elite LaChrom, Merck HITACHI, USA) coupled to a UV detector (*λ* = 205 nm). Elution was done using an ICE-COREGEL 87H3 9 µm 150 × 7.8 mm column and its pre-column (Concise Separations, San Jose, CA, USA) at 50°C with 0.04 M sulfuric acid as mobile phase (0.6 ml/min). Data were analyzed with the EZChrom Elite software. SCFA concentrations were calculated from standard curves established with a known equimolar concentration solution of acetate, propionate, and butyrate (0, 10, 25, and 40 mM).

### 2.10 Lipase inhibition assay

Lipase (type II, from porcine pancreas, Sigma, Saint-Louis, MO, USA) hydrolytic activity was assessed by monitoring the conversion of 4-methylumbelliferyl oleate (4-MUO, Sigma, Saint-Louis, MO, USA) into 4-methylumbelliferone (4-MU). The enzymatic activity was determined by measuring 4-MU (Sigma, Saint-Louis, MO, USA) fluorescence signal at 460 nm (after excitation at 355 nm) over time. A commercial reference inhibitor, orlistat (Sigma, Saint-Louis, MO, USA), was used as positive control, and citrate-phosphate buffer (0.1 M, pH 7.4) as reaction buffer. A series of 4-MU dilutions in buffer was used to generate the standard curve to convert the absorbance values into 4-MU concentrations. A 4 g/L solution of lipase in buffer was centrifuged at 5 000 g, 10°C for 10 min, then the supernatant was aliquoted. The final in-well concentration used in the assay was 250 mg/L. In each microplate well, 20 µl of test products diluted to 10:90 (v:v) in a DMSO:water mixture (or 20 µl of this solvent mixture for controls), 20 µl of lipase in buffer (or 20 µl of buffer for the blanks), and 110 µl of buffer were incubated at 37°C in a microplate reader for 10 min. Then, 10 µl of 4-MUO substrate (48 µM in DMSO) was added to each well to initiate the reaction. Fluorescence was measured at 355 nm/460 nm (excitation/emission) using a FLUO Star Omega (BMG LabTech) 96-well microplate reader, maintained at 37°C, every 30 s for 15 min. The relative lipase activity was calculated using Equation 1. The IC_50_ of each compound corresponded to the lowest concentration at which the lipase activity was halved.

(1) $$\text{\%}\;\;\mathrm{lipase}\;\;\mathrm{activity}=\frac{\mathrm{Residual}\;\mathrm{activity}}{\mathrm{Negative}\;\;\mathrm{control}\;\;\mathrm{activity}}\times 100$$

### 2.11 Lipidomics analysis

Lipidomic analysis was performed at the Center for Proteomics and Metabolomics, Leiden University, with feces collected as described above. Lipids were extracted from 50 mg of dry feces using the methyl-tert-butylether method, and analyzed using Lipidyzer™, a direct infusion-tandem mass spectrometry (DI-MS/MS)-based platform (Sciex, Redwood City, USA), as described previously (37, 38). Lipid concentrations were expressed as pmol/mg of dry feces.

### 2.12 Microbiota analysis

Fresh feces were collected during week 6 of the study and immediately frozen in liquid nitrogen. DNA was extracted from 50 to 100 mg of sample using the FastDNATM Spin Kit for feces (MP Biomedicals, Illkirch, France) and FastPrep-24 5G (MP Biomedicals, Illkirch, France) following the manufacturer's instructions. DNA was eluted in 50 µl of elution buffer, pre-heated at 55°C, and normalized to 20 ng/µl. Microbial 16S amplicon libraries were prepared at the Plateforme Génome Transcriptome de Bordeaux, Bordeaux, France, by amplification and sequencing of the V3–V4 region of the 16S rRNA amplicon on an Illumina MiSeq machine, using the 2 × 250 base pair Illumina v2 kit. Quality filtering and processing were done as described previously (39) with cutadapt v4.0 (40) and Python v3.9.9, usearch v11.0.667 (41, 42) and mothur v1.48.0 (43).Classification of quality filtered reads was performed by comparison with the ribosomal database project (RDP trainset 14) (44, 45).

### 2.13 Statistical analysis

Prism V.7.0 and 8.0 (GraphPad Software) were used for statistical tests and figure drawing. The Shapiro–Wilk normality test was used to determine whether data followed a Gaussian distribution. If data were not normally distributed, the Kruskal–Wallis nonparametric test was used followed by the Dunn test for *post hoc* comparison. When the normal distribution was confirmed, data were compared with the one-way or two-way ANOVA and Tukey's test for multiple comparisons. For measurements repeated over time, differences between groups and time points were evaluated using a repeated-measure two-way ANOVA followed by the Tukey's *post hoc* test for multiple comparisons. If missing data did not allow using the repeated-measures two-way ANOVA, a mixed-effects analysis was performed. Values are presented as the mean ± SEM, unless specified otherwise. Differences were considered significant at *p* < 0.05.

For microbiota analyses, relative abundance tables were imported and analyzed in R v4.1. Alpha (Chao and Shannon indices) and beta diversity (Bray-Curtis and Jaccard distances) were estimated using vegan 2.6–2 (46) and compared using the Kruskal-Wallis and MANOVA tests, respectively. Indicator taxa were selected using the KW test and multiple testing correction with False Discovery Rate (FDR) correction. For indicator taxa, an additional linear model was tested to determine the type of observed effect. Correlations between the relative abundances of taxa and arbitrary values were tested using the Pearson correlation coefficient. Arbitrary units were allocated to the experimental groups and six models were tested: (1) Totum-070 induces a return to normal that goes beyond the ND value (ND = 25, WD = 75; WD-T070 3 g/kg = 10), (2) Totum-070 induces a return to normal (ND = 25, WD = 75; WD-T070 3 g/kg = 25), (3) Totum-070 induces a partial return to normal (ND = 25, WD = 75; WD-T070 3 g/kg = 50), (4) Totum-070 does not rescue the changes induced by WD (ND = 25, WD = 75; WD-T070 3 g/kg = 75), (5) Totum-070 accentuates the WD effect (ND = 25, WD = 75; WD-T070 3 g/kg = 100), (6) Totum-070, but not WD, induces a change (ND = 100, WD = 100; WD-T070 3 g/kg = 10). Indicator taxa were identified by selecting taxa significantly associated with the supplementation group in the Kruskal-Wallis test, and for which at least one of the linear models was significant. If multiple linear models were significant, the model with the highest effect size was reported in Table 2. Correlations between indicator taxa and metabolic parameters were assessed using the Pearson correlation with FDR correction for multiple testing.

**Table 2:** Fecal microbiota: relative abundance differences at the phylum level in the different groups.

	% of relative abundance			Adjusted *p*. values		
Phylum	ND	WD	WD-T070 3 g/kg	ND vs. WD	ND vs. WD-T070 3 g/kg	WD vs. WD-T070 3 g/kg
Actinobacteria	8.50 ± 5.43	0.99 ± 0.40	1.22 ± 1.05	0.0014	0.0085	0.9292
Firmicutes	56.03 ± 5.35	59.16 ± 4.86	53.07 ± 8.78	0.4500	0.9342	0.8885
Bacteroidetes	26.14 ± 4.44	30.16 ± 6.59	32.78 ± 4.11	0.4500	0.0214	0.3490
Verrucomicrobia	3.42 ± 4.31	2.59 ± 4.38	4.76 ± 6.62	0.4500	0.5654	0.3490
uncl. Bacteria	2.40 ± 0.45	2.11 ± 0.18	2.23 ± 0.48	0.1318	0.5654	0.7574
Deferribacteres Candidatus	1.88 ± 3.92	2.63 ± 4.71	1.43 ± 2.38	0.4500	0.5654	0.3490
Saccharibacteria	0.24 ± 0.19	0.29 ± 0.36	0.56 ± 0.70	0.9438	0.5654	0.8032
Campilobacterota	0.00 ± 0.00	0.00 ± 0.01	0.00 ± 0.00	0.9438	0.5654	0.6680
Tenericutes	0.06 ± 0.09	0.00 ± 0.00	0.00 ± 0.00	0.0405	0.0408	
Proteobacteria	1.33 ± 1.04	2.07 ± 1.29	3.93 ± 1.38	0.1318	0.0085	0.0227
F/B ratio	2.22 ± 0.57	2.06 ± 0.51	1.67 ± 0.45	0.5262	0.0390	0.1309

Relative abundances are presented as the mean ± SD, and *p* values were adjusted for multiple testing (*n* = 11 ND, *n* = 10 WD, *n* = 8 WD- T070 3 g/kg). F/B, firmicutes/bacteroidetes.

## 3 Results

### 3.1 Totum-070 supplementation limits body weight gain in WD-fed mice

**Figure 1:**
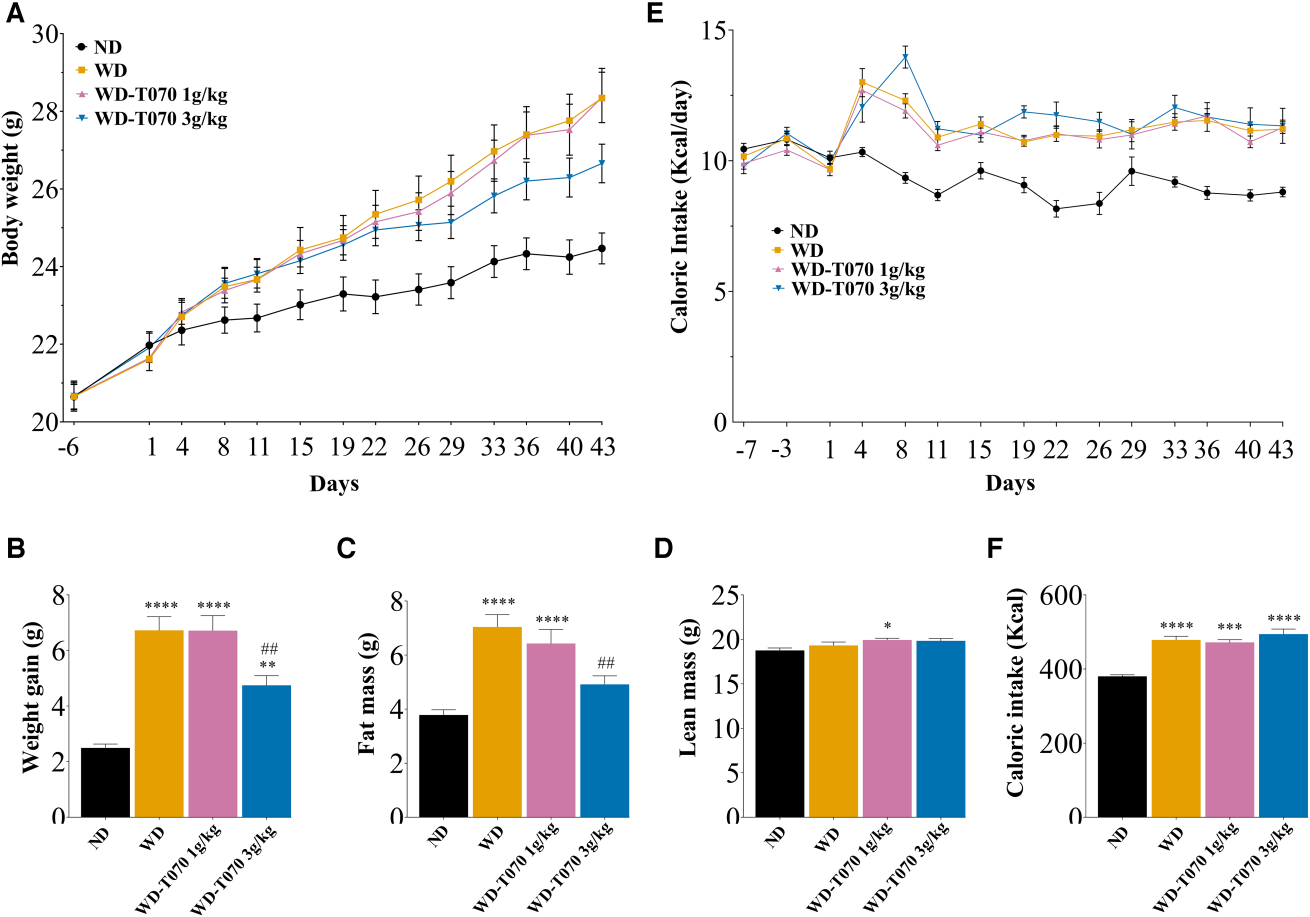
Effect of supplementation with Totum-070 (1 g/kg and 3 g/kg) in Western diet-fed mice on body weight, body composition and caloric intake during the study period. (**A**) Starting at day 1, male C57BL/6JOlaHsd mice were fed a normal diet (ND), a Western diet (WD) or a WD plus daily gavage with Totum-070 (WD-T070, 1 g/kg or 3 g/kg) (*n* = 14 per group). Body weight was recorded during the study. (**B**) Body weight gain (difference between the last body weight at day 43 and the body weight at the study start, day 1). (**C**) Fat mass and (**D**) lean mass measured by EchoMRI at the study end. (**E**) Caloric intake during the study period. (**F**) Cumulative caloric intake during the study. * *p* < 0.05, ** *p* < 0.01, and *** *p* < 0.001 vs. ND. ## *p* < 0.01 Totum-070 groups vs. WD. Data are the mean ± SEM.

To establish a model of lipid metabolic disorder, mice were fed a WD (*n* = 42) or a normal diet (ND; *n* = 14) for 6 weeks. Animals fed a WD were subdivided in three groups: WD alone (WD; *n* = 14), WD supplemented with 1 g/kg of Totum-070 (WD-T070 1 g/kg; *n* = 14), and WD supplemented with 3 g/kg of Totum-070 (WD-T070 3 g/kg; *n* = 14). Totum-070 (or vehicle alone in the WD and ND groups) was administered by gavage daily. The final body weight was significantly higher in the WD groups than ND group (Figure 1A). Body weight was similar between the WD and WD-T070 1 g/kg groups throughout the study (Figures 1A,B). Conversely, after 4 weeks of supplementation, body weight increase was reduced in the WD-T070 3 g/kg group compared with WD mice (Figure 1A). This resulted in a 30% lower (*p* < 0.01) body weight gain (Figure 1B) and fat mass gain (Figure 1C) compared with the WD group at the supplementation end. No change was observed in lean mass between the WD group and mice supplemented with Totum-070 (both concentrations) (Figure 1D). During the study, the daily caloric intake was higher in the WD groups compared with the ND group (Figure 1E), and was similar among the WD groups (WD and WD-T070) (Figures 1E,F). Therefore, Totum-070 gavage did not affect energy intake in WD mice.

### 3.2 Totum-070 supplementation prevents cholesterol elevation in WD-fed mice

**Figure 2:**
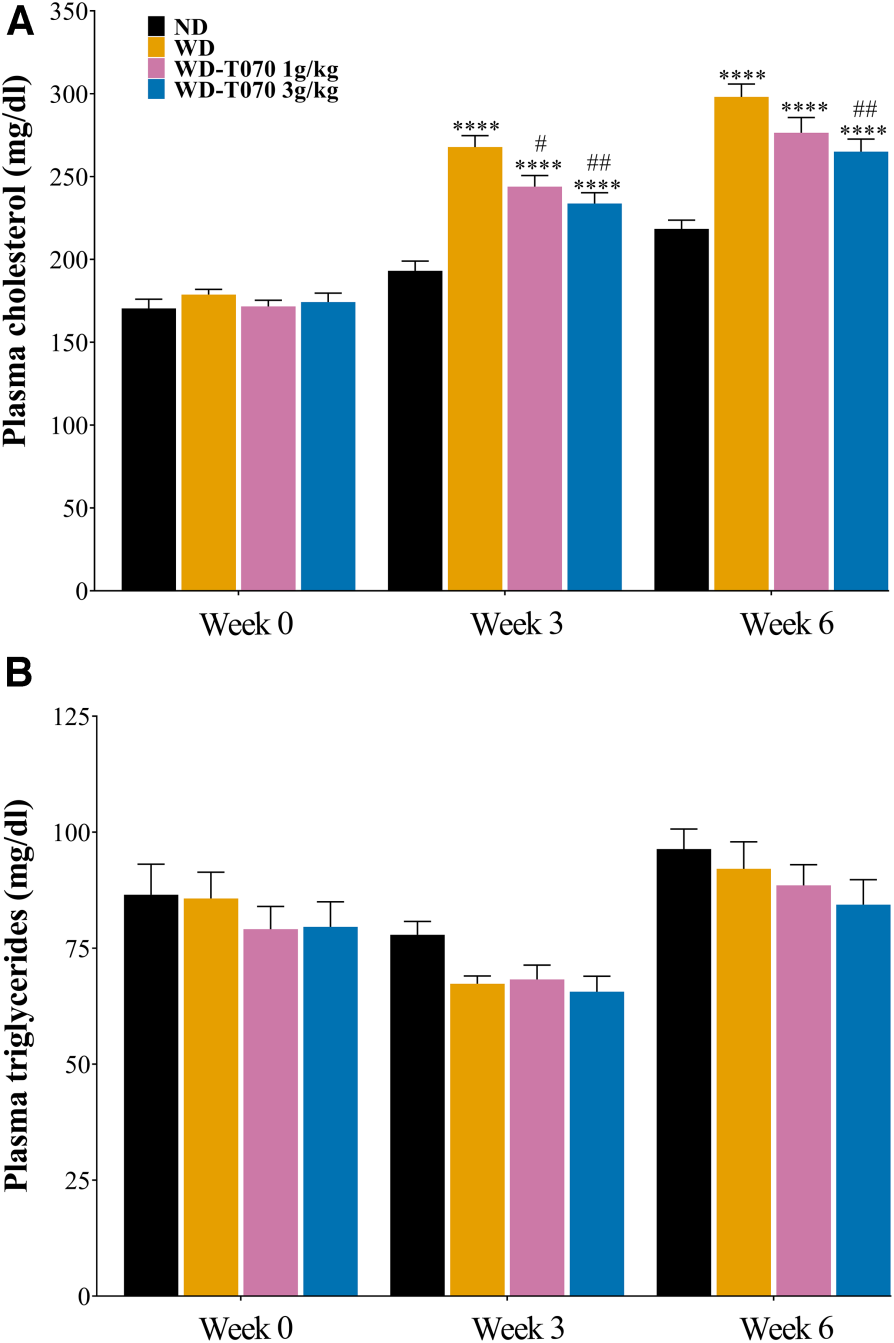
Effect of Totum-070 supplementation on plasma lipid concentration. (**A**) Plasma concentration of cholesterol (*n* = 14 per group) and (**B**) of triglycerides (*n* = 14 per group) at week 0 (before starting the WD), during the study (week 3), and at the study end (week 6). **** *p* < 0.0001 vs. ND. # *p* < 0.05 and ## *p* < 0.01 Totum-070 (T070) groups vs. WD. Data are the mean ± SEM.

At the study start (week 0), plasma cholesterol concentrations were identical in all groups (Figure 2A). After 3 weeks, plasma cholesterol was significantly increased (+38%) in the WD group, and this level was maintained at the study end (+36%) (Figure 2A). Totum-070 (1 g/kg and 3 g/kg) dose-dependently reduced plasma cholesterol concentration after 3 weeks (−8.7%, *p* < 0.05 and −12.7%, *p* < 0.01 vs. WD group, respectively), and after 6 weeks of supplementation (−7.3%, *p* = 0.08 and −11%, *p* < 0.01 vs. WD group, respectively) (Figure 2A). Circulating triglyceride concentration was similar in all groups throughout the study (Figure 2B).

### 3.3 Totum-070 supplementation improves hepatic lipid metabolism

**Figure 3:**
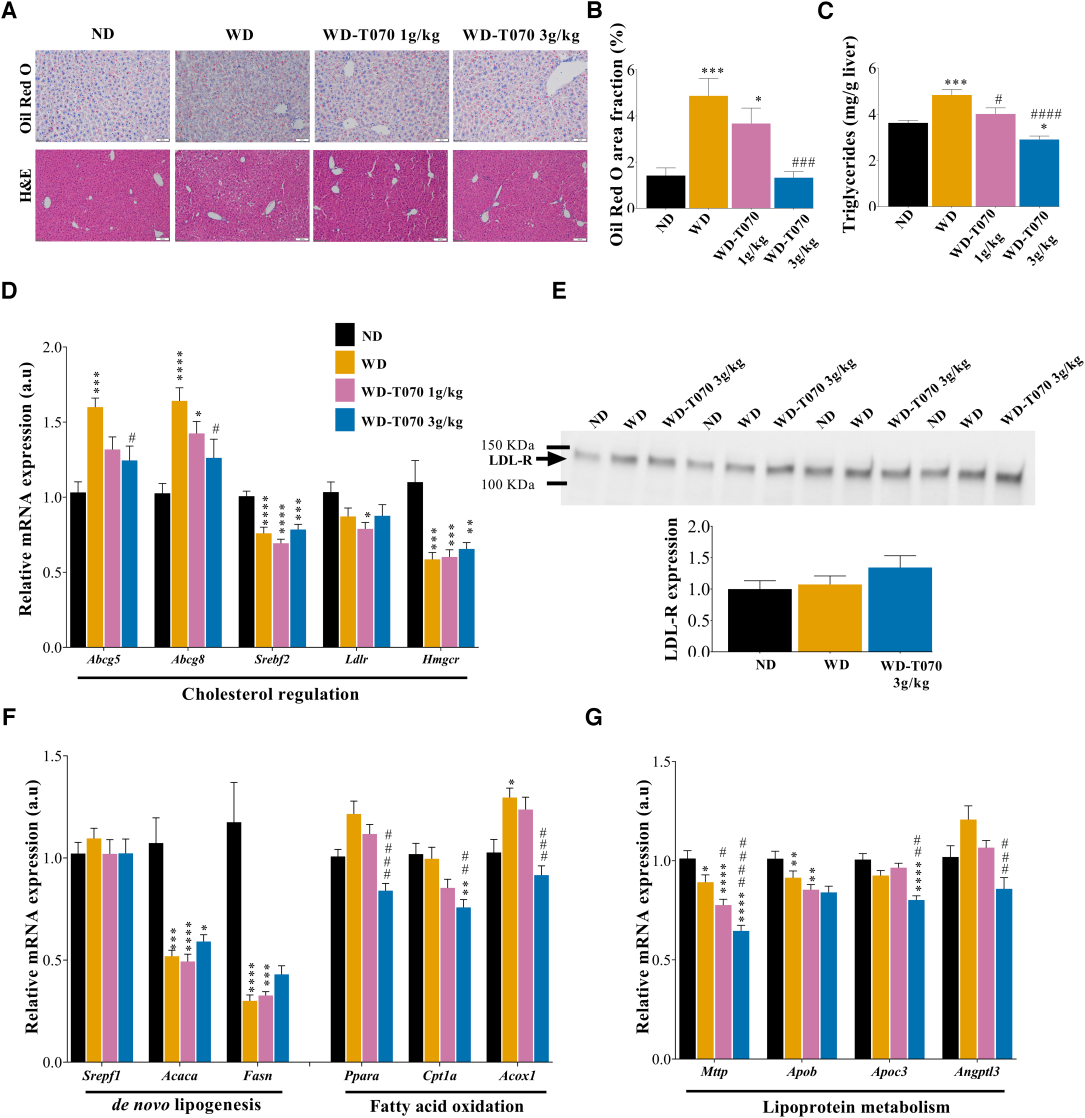
Effect of Totum-070 on liver lipid metabolism. (**A**) Frozen mouse liver tissue sections stained with Oil Red O and counterstained with hematoxylin (upper panels). Scale bars, 50 µm. Paraffin-embedded sections of mouse livers stained with hematoxylin and eosin (H&E) (lower panels). Scale bar, 100 µm. (**B**) Quantification of Oil Red O in liver sections (at least three images per liver, *n* = 14 per group) shows reduction of neutral lipid accumulation in mice supplemented with Totum-070. **(C**) Quantification of hepatic triglyceride content (*n* = 14 per group). (**D**) Relative expression in liver of selected genes encoding proteins implicated in cholesterol metabolism (*n* = 14 per group). (**E**) Immunoblot to monitor LDL receptor (LDL-R) expression in liver samples from the indicated groups and quantification. (**F**) Relative expression in mouse liver samples from the indicated groups of selected genes encoding proteins implicated in lipid metabolism. (**G**) Relative expression in mouse liver samples from the indicated groups of selected genes encoding proteins implicated in lipoprotein metabolism. *p < 0.05, **p < 0.01, ***p < 0.001 and ****p < 0.0001 vs. ND. #*p* < 0.05, ##*p* < 0.01, ###*p* < 0.001 and ####*p* < 0.001 Totum-070 (T070) groups vs. WD. Data are the mean ± SEM.

Histological analysis of liver samples revealed abnormal accumulation of lipid droplets induced by WD that was reduced by Totum-070 supplementation (Figure 3A). In agreement, quantification of Oil Red O staining in liver sections confirmed in the WD-T070 3 g/kg group, the reduction of neutral lipid accumulation to the level observed in the ND group (Figure 3B). Moreover, hepatic triglyceride content was reduced by 17% (*p* < 0.05) in the WD-T070 1 g/kg group and by 40% (*p* < 0.0001) in the WD-T070 3 g/kg group (Figure 3C) compared with the WD group. Analysis of the expression of genes implicated in liver lipid and lipoprotein metabolism showed that Totum-070 supplementation influenced the expression of some genes involved in cholesterol metabolism (Figure 3D). Specifically, Totum-070 supplementation limited the upregulation of the cholesterol transporters *Abcg5* and *Abcg8* induced by the WD. Conversely, it did not have any effect on the expression levels of *Srebf2* and the downstream genes *Ldlr* and *Hmgcr* that were decreased in all WD groups. WD and Totum-070 supplementation did not have any effect on LDL receptor (LDL-R) protein expression in liver (Figure 3E). Totum-070 supplementation did not have any effect on the expression of genes implicated in *de novo* lipogenesis, whereas it decreased (vs. WD) the expression of genes involved in fatty acid oxidation (*Ppara*, *Cpt1a*, *Acox1*), but only when used at the concentration of 3 g/kg (Figure 3F). Totum-070 further decreased the expression of genes involved in lipoprotein metabolism, such as *Mttp* (both doses compared with the WD and ND groups) and also *Apoc3* and *Angptl3*, but only at the higher dose (Figure 3G).

### 3.4 Totum-070 modulates the intestinal lipid metabolism

**Figure 4:**
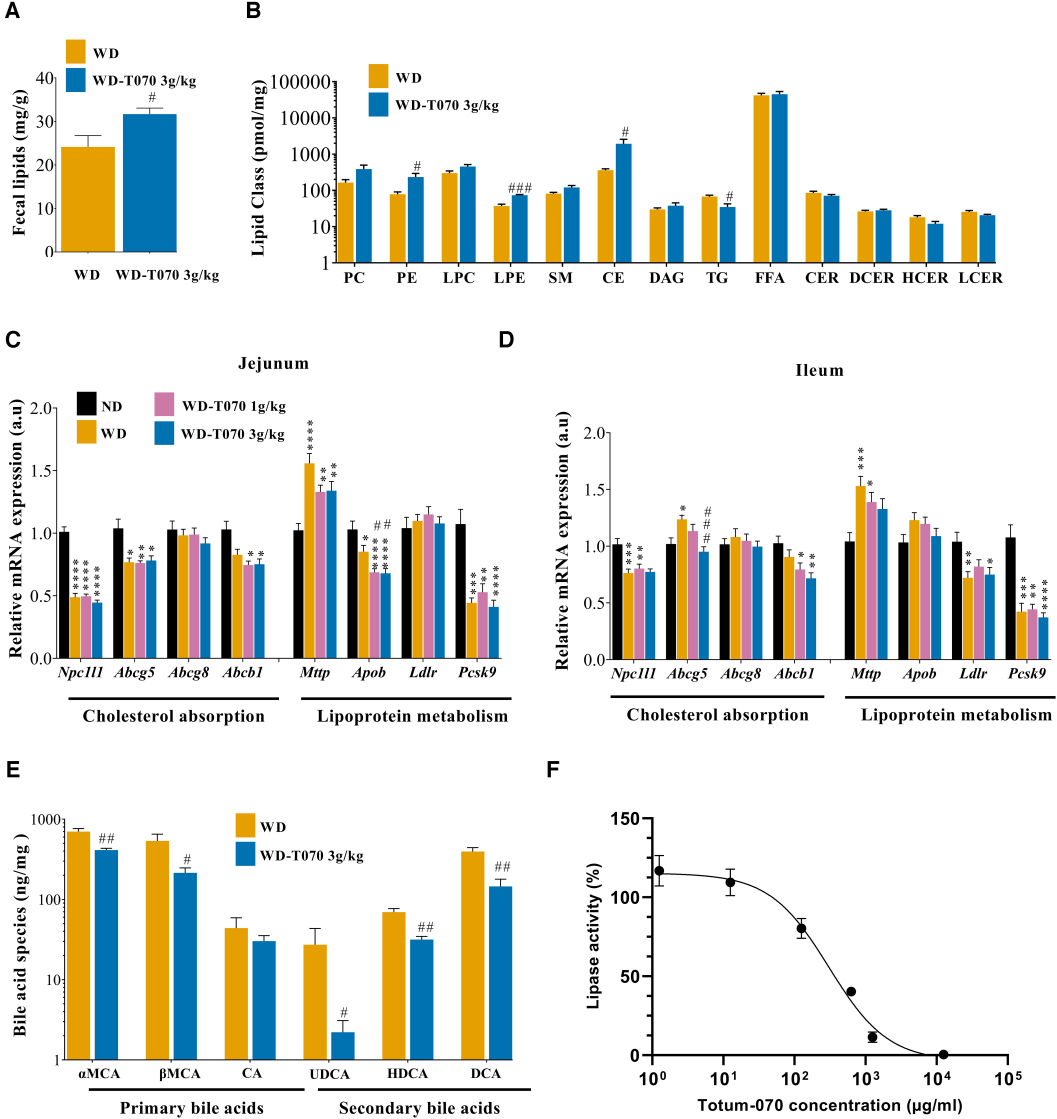
Effect of Totum-070 on intestinal lipid metabolism. (**A**) Total lipid content in feces (*n* = 5 per group). (**B**) Quantification of different lipid classes in feces. CE, cholesterol ester; CER, ceramides; DAG, diacylglycerides; DCER, dihydroceramides; FFA, free fatty acids; HCER, hexosylceramides; LPC, lysophosphatidylcholine; LPE, lysophosphatidylethanolamine; PC, phosphatidylcholine; PE, phosphatidylethanolamine; SM, sphingomyelin; and TG, triacylglycerides. (**C,D**) Relative expression of selected genes implicated in cholesterol and lipoprotein metabolism in jejunum (**C**), and ileum samples (**D**) (*n* = 14 samples per tissue and per group). (**E**) Quantification of different bile acid classes in feces (*n* = 5 per group). αMCA, α-muricholic acid; βMCA, β-muricholic acid; CA, cholic acid; UDCA, ursodeoxycholic acid; HDCA, hyodeoxycholic acid; DCA, deoxycholic acid. (**F**) Inhibition of lipase activity by Totum-070, *n* = 4–11 replicates. *p < 0.05, **p < 0.01, ***p < 0.001 and ****p < 0.0001 vs. ND. #*p* < 0.05, ##*p* < 0.01, ###*p* < 0.001 and ####*p* < 0.0001 Totum-070 (T070) groups vs. WD. Data are the mean ± SEM.

To better understand the regulation of lipid homeostasis by Totum-070, total lipid content was quantified in feces from mice in the WD and WD-T070 3 g/kg groups (Figure 4A). This showed that fecal lipid content was increased by 31% in the supplemented group (*p* < 0.05). Lipidomic analyses in stool samples highlighted the increase of specific lipid classes in the WD-T070 3 g/kg group compared with the WD group: cholesterol ester (CE) (+437%, *p* < 0.05), lysophosphatidylethanolamine (LPE) (+98%, *p* < 0.001) and phosphatidylethanolamine (PE) (+197%, *p* < 0.05). Conversely, triglycerides (TG) were decreased by 49% (*p* < 0.05) in feces from the WD-T070 3 g/kg group compared with the WD group (Figure 4B). Genes implicated in intestinal cholesterol absorption were assessed for differential expression between groups in the duodenum (Supplementary Figure S1A), jejunum (Figure 4C) and ileum (Figure 4D). Most investigated genes were not influenced by Totum-070 in the three intestinal segments. Apob mRNA level was repressed specifically in the jejunum (Figure 4C). The transcript for Abcg5 was downregulated by Totum-070 in the ileum only (Figure 4D).

To complete the characterization of pathways linked to cholesterol metabolism, the concentration of the major bile acid species was measured in feces. With the exception of cholic acid (CA), all bile acid classes were significantly reduced in feces from mice in the WD-T070 3 g/kg group compared with the WD group: α-muricholic acid (*α*MCA) −41%; β-muricholic acid (βMCA) −60%; ursodeoxycholic acid (UDCA) −92%; hyodeoxycholic acid (HDCA) −54.5%; and deoxycholic acid (DCA) −63% (Figure 4E). Based on these data, the expression of genes associated with bile acid regulation was assessed in liver and ileum (Supplementary Figure S1B,C). In liver, *Cyp8b1* and *Cyp27a1* were significantly downregulated in the WD-T070 3 g/kg group compared with the WD group as well as *Slc10a1* and *Slco1a5*, which encode transporters implicated in the regulation of bile acid uptake by hepatocytes. In ileum, none of the genes investigated was affected by Totum-070 administration.

Lastly, an *in vitro* pancreatic lipase activity inhibition test was used to directly assess Totum-070 effect on intestinal lipid digestion. Comparison of Totum-070 and orlistat (the reference lipase inhibitor drug) effects on lipase activity (Figure 4F and Supplementary Figure S1D) showed that Totum-070 inhibited lipase activity (IC_50_ = 305.6 µg/ml), but not as strongly as orlistat (IC_50_ = 0.0084 µg/ml.

### 3.5 Effect of Totum-070 supplementation on fecal microbiota

**Figure 5:**
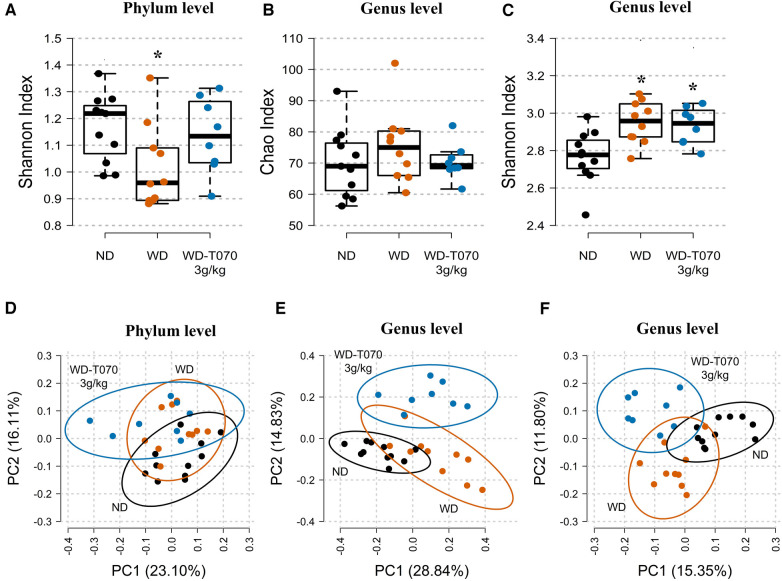
Effect of Totum-070 on richness, diversity and composition of fecal microbiota. (**A**) Shannon diversity index at the phylum level. (**B**) Rarefied Chao index (richness index) at the genus level. (**C**) Shannon diversity index at the genus level. (**D**) and (**E**) Principal coordinate analysis based on the Bray–Curtis dissimilarity index at the phylum and genus levels, with 95% confidence standard deviation ellipses. (**F**) Principal coordinate analysis based on the Jaccard dissimilarity index at the genus level, with 95% confidence standard deviation ellipses. Boxplots show the median, 25th and 75th percentile, and adjacent values. *n* = 11 ND, *n* = 10 WD, *n* = 8 WD-T070 3 g/kg. * *p* < 0.05 vs. ND.

**Figure 6:**
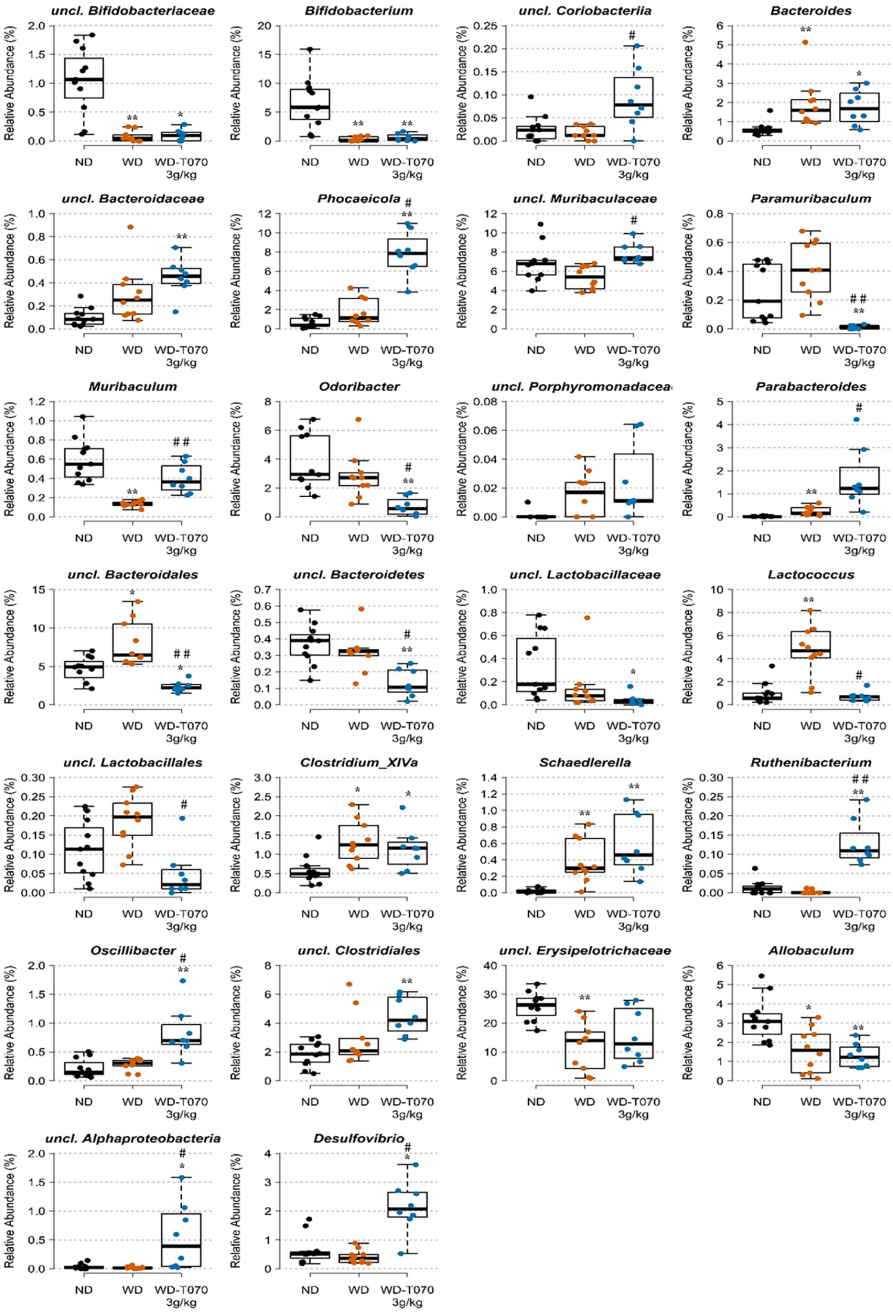
Effect of Totum-070 on composition of fecal microbiota at the genus level. Figures represent relative abundance values of genera that differed significantly between two groups or more. Boxplots show the median, 25th and 75th percentile, and adjacent values. *n* = 11 ND, *n* = 10 WD, *n* = 8 WD-T070 3 g/kg. *p < 0.05, **p < 0.01, ***p < 0.001 and ****p < 0.0001 vs. ND. #*p* < 0.05, ##*p* < 0.01, ###*p* < 0.001 and ####*p* < 0.0001 Totum-070 (T070) group vs. WD.

Next, 16S rRNA amplicon sequencing was used to identify the microbiota structure in feces collected from mice in the ND, WD and WD-T070 3 g/kg groups at the study end. At the phylum level, evenness (Shannon index) was significantly decreased in the WD group (*p* < 0.05), compared with the ND group, but not in the WD-T070 3 g/kg group (Figure 5A). At the genus level, species richness (Chao index) was comparable among groups, whereas evenness (Shannon index) was increased in both WD and WD-T070 3 g/kg groups compared with the ND group (Figure 5B,C). Principal coordinate analysis of the relative abundance of intestinal microbial communities at the phylum level using the Bray-Curtis dissimilarity index followed by PERMANOVA showed that microbial composition differed significantly in the three groups (Bray-Curtis, adonis: *p* < 0.05 R_NDvsWD_ = 016, *p* < 0.01 R_NDvsWD−T070 3 g/kg_ = 0.24, *p* < 0.1 R_WDvsWD−T070 3 g/kg_ = 0.12) (Figure 5D). The same analysis at the genus level confirmed that the WD strongly affected microbial composition compared with the ND (Bray-Curtis, adonis: *p* < 0.0001, R_NDvsWD_ = 0.32; Figure 5E and Jaccard, adonis: *p* < 0.0001, R_NDvsWD_ = 0.20; Figure 5F). The microbial composition also differed between the WD-070 group and the ND group (Bray-Curtis, adonis: *p* < 0.0001 R_WDvsWD−T070 3 g/kg_ = 0.39; Figure 5E and Jaccard, adonis: *p* < 0.0001, R_WDvsWD−T070 3 g/kg_ = 0.21; Figure 5F) and also the WD group (Bray-Curtis, adonis: *p* < 0.001, R_WDvsWD−T070 3 g/kg_ = 0.26; Figure 5E and Jaccard, adonis: *p* < 0.0001, R_WDvsWD−T070 3 g/kg _= 0.20; Figure 5F). Given the supplementation effect on the alpha and beta diversity measures and the visible differences in the average composition at the phylum and genus levels, an indicator species analysis was performed to identify the taxa that were specifically affected in the different conditions. Data are shown at the phylum level (Table 2) and genus level (Figure 6 and Table 3) as results of the two extremes of the taxonomic levels.

**Table 3:** Fecal microbiota: relative abundance differences at the genus level.

Genus	% of relative abundance			Adjusted *p*. values			Plasma cholesterol	
	ND	WD	WD-T070 3 g/kg	ND vs. WD	ND vs. WD-T070 3 g/kg	WD vs. WD-T070 3 g/kg	Person	Adjusted *p*. values
uncl. *Bifidobacteriaceae*	1.05 ± 0.58	0.07 ± 0.10	0.10 ± 0.10	0.0067	0.0133	0.5270	−59.4%	0.0058
*Bifidobacterium*	6.63 ± 4.43	0.30 ± 0.38	0.57 ± 0.62	0.0065	0.0099	0.4050	−55.4%	0.0119
uncl. *Coriobacteriia*	0.03 ± 0.03	0.02 ± 0.01	0.09 ± 0.07	0.7760	0.0652	0.0360	4.6%	0.8128
*Bacteroides*	0.61 ± 0.35	1.93 ± 1.26	1.74 ± 0.89	0.0067	0.0133	1.0000	52.9%	0.0137
uncl. *Bacteroidaceae*	0.10 ± 0.08	0.30 ± 0.24	0.45 ± 0.16	0.0598	0.0065	0.1645	46.4%	0.0322
*Phocaeicola*	0.67 ± 0.52	1.71 ± 1.36	7.79 ± 2.29	0.1621	0.0058	0.0111	32.1%	0.1549
uncl. *Muribaculaceae*	6.86 ± 1.95	5.39 ± 1.20	7.85 ± 1.06	0.1636	0.2233	0.0162	−29.0%	0.1998
*Paramuribaculum*	0.25 ± 0.20	0.41 ± 0.20	0.01 ± 0.01	0.2441	0.0058	0.0096	18.3%	0.4222
*Muribaculum*	0.60 ± 0.22	0.13 ± 0.03	0.40 ± 0.15	0.0065	0.2086	0.0096	65.9%	0.0026
*Odoribacter*	3.78 ± 1.89	2.85 ± 1.61	0.70 ± 0.62	0.2910	0.0065	0.0181	−21.8%	0.3337
uncl. *Porphyromonadaceae*	0.00 ± 0.00	0.02 ± 0.02	0.02 ± 0.03	0.0590	0.0084	0.6573	47.5%	0.0322
*Parabacteroides*	0.02 ± 0.02	0.24 ± 0.18	1.64 ± 1.29	0.0065	0.0058	0.0162	23.5%	0.2998
uncl. *Bacteroidales*	4.65 ± 1.58	7.94 ± 2.92	2.38 ± 0.66	0.0463	0.0133	0.0096	35.8%	0.1074
uncl. *Bacteroidetes*	0.37 ± 0.12	0.32 ± 0.12	0.13 ± 0.08	0.3998	0.0089	0.0181	−11.9%	0.6376
uncl. *Lactobacillaceae*	0.34 ± 0.28	0.15 ± 0.22	0.04 ± 0.05	0.1636	0.0133	0.1983	−38.9%	0.0800
*Lactococcus*	0.98 ± 0.92	4.66 ± 2.19	0.73 ± 0.43	0.0074	0.7202	0.0116	46.6%	0.0322
uncl. *Lactobacillales*	0.11 ± 0.08	0.19 ± 0.07	0.05 ± 0.06	0.1452	0.1298	0.0193	9.7%	0.6663
*Clostridium_XlVa*	0.58 ± 0.36	1.32 ± 0.54	1.14 ± 0.54	0.0246	0.0442	0.6573	45.3%	0.0354
*Schaedlerella*	0.02 ± 0.03	0.38 ± 0.26	0.60 ± 0.36	0.0067	0.0058	0.3201	35.6%	0.1074
*Ruthenibacterium*	0.01 ± 0.02	0.00 ± 0.00	0.13 ± 0.06	0.1921	0.0058	0.0096	10.3%	0.6663
*Oscillibacter*	0.22 ± 0.16	0.28 ± 0.10	0.83 ± 0.43	0.4392	0.0080	0.0181	26.6%	0.2361
uncl. *Clostridiales*	1.87 ± 0.87	2.84 ± 1.78	4.49 ± 1.28	0.4392	0.0058	0.0974	44.6%	0.0361
uncl. *Erysipelotrichaceae*	25.87 ± 4.88	11.79 ± 8.28	15.50 ± 9.24	0.0067	0.1998	0.4050	−62.9%	0.0034
*Allobaculum*	3.18 ± 1.13	1.58 ± 1.14	1.31 ± 0.62	0.0416	0.0099	0.7845	−53.4%	0.0137
uncl. *Alphaproteobacteria*	0.03 ± 0.05	0.02 ± 0.02	0.55 ± 0.58	0.7760	0.0302	0.0199	28.7%	0.1998
*Desulfovibrio*	0.64 ± 0.50	0.42 ± 0.23	2.14 ± 0.89	0.4392	0.0133	0.0116	6.2%	0.7791

Indicator taxa were identified as those with relative abundance values that differed significantly between two groups or more. Relative abundances are presented as mean ± SD and *p* values were adjusted for multiple testing. Correlations between indicator taxa and plasma total cholesterol was assessed using the Pearson correlation with FDR correction for multiple testing (*n* = 11 ND, *n* = 10 WD, *n* = 8 WD-T070 3 g/kg).

At the phylum level, supplementation with Totum-070 (3 g/kg) led to an increase in the relative abundance of Proteobacteria compared with the WD group (+89%, *p* < 0.05) and the ND group (+195%, *p* < 0.01). Moreover, the Firmicutes to Bacteroidetes ratio remained identical between WD and ND (Table 2) but was 20.5% lower in the WD-T070 3 g/kg group compared with the ND group (*p* < 0.05).

At the genus level (Figure 6 and Table 3), the proportions of uncl. *Coriobacteriia* (*p* = 0.036), *Phocaeicola* (*p* = 0.0111), uncl. *Muribaculaceae* (*p* = 0.0162), *Muribaculum* (*p* = 0.0096), *Parabacteroides* (*p* = 0.0162), *Ruthenibacterium* (*p* = 0.0096), *Oscillibacter* (*p* = 0.0181), uncl. *Alphaproteobacteria* (*p* = 0.0199) and *Desulfovibrio* (*p* = 0.0116) were significantly increased in the WD-T070 3 g/kg group compared with the WD group. Conversely, *Paramuribaculum* (*p* = 0.0096), *Odoribacter* (*p* = 0.0181), uncl. *Bacteroidales* (*p* = 0.0096), uncl. *Bacteroidetes* (*p* = 0.0181), *Lactococcus* (*p* = 0.0116) and uncl. *Lactobacillales* (*p* = 0.0193) were significantly decreased in the WDT070 3 g/kg group compared with the WD group. Noteworthy, some WD-linked alterations were reversed by Totum-070 supplementation. Specifically, *Muribaculum* richness was significantly reduced in WD-fed mice (−79%, *p* < 0.01, WD vs. ND), but was increased by Totum-070 (+207%, *p* < 0.01, WD-T070 3 g/kg vs. WD). Moreover, the Pearson's correlation analysis showed a significant negative association between *Muribaculum* richness and plasma cholesterol (*p* = 0.0026). Conversely, *Lactococcus* richness was significantly increased by WD (+375%, *p* < 0.01, WD vs. ND, respectively) and reduced by Totum-070 supplementation (−84%, *p* < 0.05, WD-T070 3 g/kg vs. WD). The relative abundance of *Lactococcus* was positively correlated with plasma cholesterol (*p* = 0.0322) (Table 3).

### 3.6 The hypocholesterolemic effect of totum-070 is associated with increased microbiota-derived SFCAs

**Figure 7:**
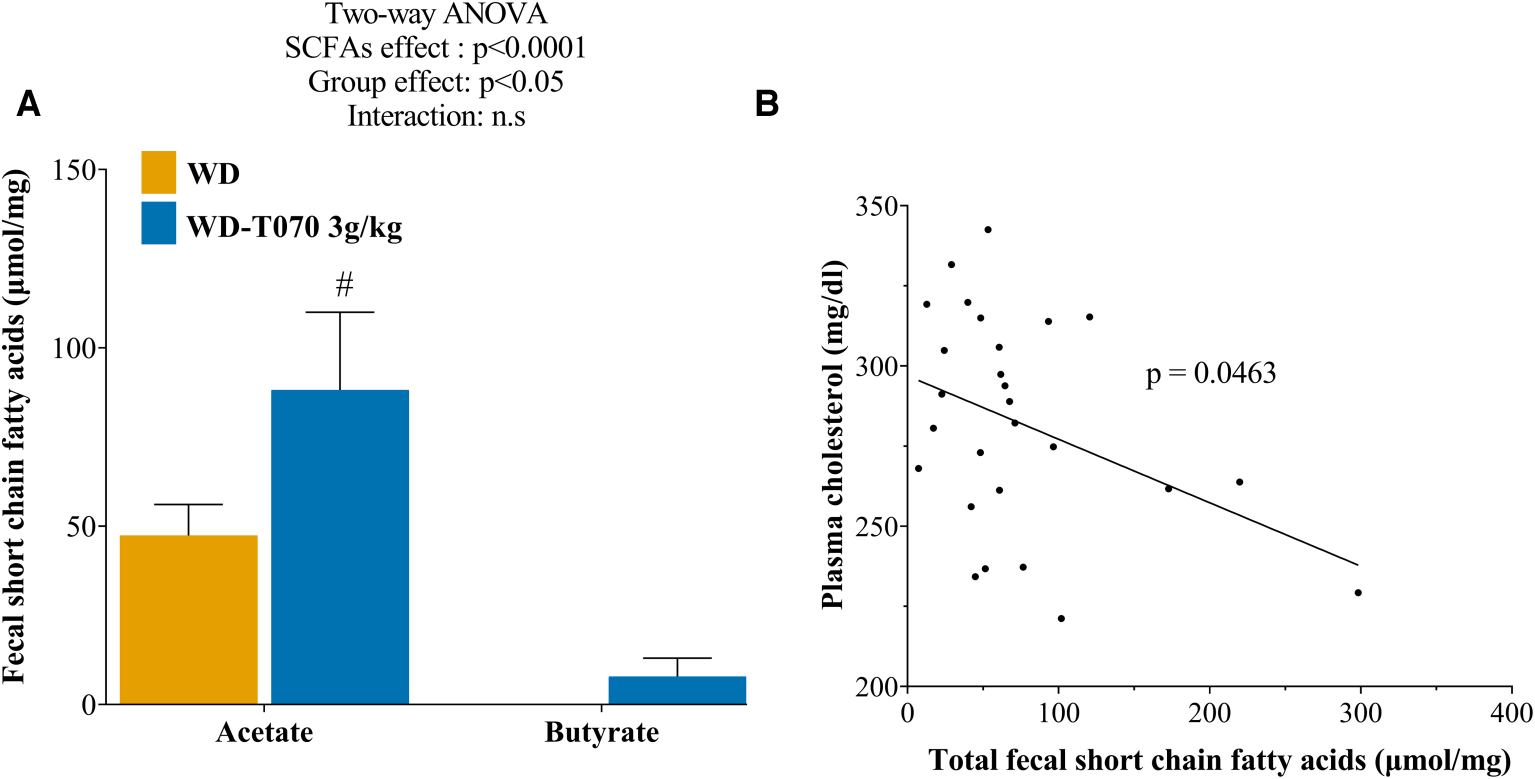
Effect of Totum-070 on fecal short chain fatty acid (SCFA) composition. (**A**) Quantification of SCFA species content in feces (*n* = 14 per group) at the study end. (**B**) Relationship between plasma cholesterol levels and fecal SCFA content in the WD and WD-T070 3 g/kg groups. #*p* < 0.05, Totum-070 (T070) group vs. WD. Data are the mean ± SEM.

Our analysis showed that the cholesterol-lowering effect of Totum-070 was related to gut microbiota modifications. As fibers and some herb-derived polysaccharides are fermented into SCFAs (mainly acetate, propionate, and butyrate) by intestinal bacteria (47) with potential beneficial effects on lipid disorders, Totum-070 effect on SCFA production was investigated at the study end. Quantification of SCFA species in the feces revealed a significant global increase of the total fecal SCFA amount in the WD-T070 3 g/kg group compared with the WD group (+102.8%, *p* < 0.05). This effect was mainly driven by fecal acetate production (Figure 7A). Post-hoc analysis indicated a significant increase of acetate in feces from WD-T070 3 g/kg mice compared with WD mice (+86%, *p* < 0.05). Propionate was not detected in any sample and butyrate only in samples from WD-T070 3 g/kg mice. The total SCFA amount in feces was negatively correlated with plasma cholesterol levels in the WD and WD-T070 3 g/kg groups (Figure 7B, *p* < 0.05).

## 4 Discussion

Atherosclerotic cardiovascular disease is the main cause of mortality worldwide (48, 49). Increased plasma low density lipoprotein cholesterol (LDL-C) level is the primary risk factor for the development of coronary heart disease and atherosclerosis (50, 51). Totum-070 combines five plant extracts involved in the regulation of cholesterol homeostasis: artichoke leaves (52–55), olive leaves (56–59), chrysanthellum (20), goji berries (60, 61) and black pepper (62, 63). Its chemical characterization revealed a high polyphenol content (>15% dry weight, Table 1). Plant-derived polyphenols are considered promising strategies to prevent metabolic disorders such as obesity, type 2 diabetes, and dyslipidemia (64, 65). Here, we evaluated the effects of Totum-070 on cholesterol levels in WD-fed hypercholesterolemic mice. After 3 weeks of supplementation, Totum-070 limited cholesterol elevation in WD-fed mice in a dose-dependent manner. These results are consistent with a recent study in dyslipidemic hamsters showing decreased total cholesterol following daily administration of 3 g/kg Totum-070 by gavage for 12 weeks (21). We also found that 3 g/kg of Totum-070 inhibited body weight and fat mass gain, whereas upon supplementation with only 1 g/kg, cholesterol reduction was not fully related to reduced weight gain. Some of the plants in Totum-070 formulation have already shown similar effects on cholesterol reduction without affecting body weight gain (56–59). In our study, total lipid content in feces from Totum-070 supplemented mice was significantly increased by 31% compared with WD-fed animals (Figure 4A). This result suggests impaired intestinal lipid absorption following Totum-070 administration that could explain, at least in part, its cholesterol-lowering effect. This hypothesis is supported by several *in vivo* and *in vitro* studies demonstrating that artichoke leaves (66) and piperine (67–69) and luteolin (70) (two active molecules present in Totum-070) inhibit intestinal cholesterol absorption. Then, we characterized the lipid-related gene response in the intestine after 6 weeks of supplementation (Figures 4C,D), but we could not establish a clear Totum-070-related pattern. Indeed, the supplementation effect on the expression of the selected genes was limited and often not conserved in the three gut segments. A chronic action of Totum-070 on intestinal gene regulation cannot be excluded. Nevertheless, phenolic compounds display very potent inhibitory effects on digestive enzymes that might explain the increased lipid fecal excretion (71). Antilipemic drugs, including orlistat, act as lipase inhibitors and are used in clinic to prevent obesity and hyperlipidemia by decreasing the dietary fat digestion and absorption in blood and by increasing fat excretion in the feces (72). Here, we found that Totum-070 inhibited pancreatic lipase activity (IC_50_ = 305.6 µg/ml, Figure 4F). This Totum-070 inhibitory effect was comparable to previous results obtained by testing different plant extracts (IC_50_ > 100 µg/ml), but was lower than the effect observed with orlistat (73–75). Literature data indicate that the artichoke and olive leaf extracts used in Totum-070 have inhibitory effects on digestive enzymes (76, 77). Several polyphenols found in Totum-070 also display inhibitory effects on pancreatic lipase, such as chlorogenic acid, oleanolic acid, di-caffeoylquinic acids, luteolin-7-O-glucoside, luteolin and apigenin (78–81). Although, it is difficult to identify the compounds responsible for a given effect, we can speculate that the inhibition of digestive enzymes by Totum-070 molecules may play a role in the hypocholesterolemic effect and in the weight gain reduction observed at the highest dose (3 g/kg body weight).

Given the importance of liver in lipid and lipoprotein regulation, we quantified the expression of selected genes to determine Totum-070 effects on liver metabolic pathways (Figure 3). Totum-070 did not have any effect on the SREBP2 signaling pathway (*Hmgcr* and *Ldlr* expression) and also on hepatic LDL-R protein expression. These results suggest that Totum-070 cholesterol lowering effect does not occur through the liver SREBP2 pathway and LDL-R-mediated LDL-C clearance. This is in contrast with the study by Wauquier et al. who found decreased *Hmgcr* expression and HMGCR enzyme activity in human HepG2 hepatocytes incubated with human sera enriched in Totum-070 metabolites (21). This discrepancy could be explained by the fact that *Hmgcr* downregulation by Totum-070 metabolites occurred in a palmitate-induced *Hmgcr* expression background in cell cultures. Conversely, our mice were fed a WD enriched in cholesterol that inhibited SREPB2 signaling, as attested by *Hmgcr* and *Ldlr* downregulation in livers from WD animals compared with the ND group. Cholesterol is eliminated from the body via its conversion into bile acids in the liver through a classic and an alternative pathway (82). In the intestine, 95% of bile acids are reabsorbed by ileum and return to liver. Therefore, bile acid sequestration to prevent their intestinal re-absorption will stimulate bile acid production from cholesterol in liver, and consequently reduce blood cholesterol level. To test whether Totum-070 cholesterol lowering effect was mediated by increased bile acid excretion, bile acid concentrations were quantified in fecal samples. Bile acid concentration was significantly reduced in feces from WD-T070 mice compared with WD mice (Figure 4E). This is in contrast with literature data showing that some Totum-070 components enhance fecal excretion of bile acids. In golden Syrian hamsters fed a HFD associated with 0.24 g/kg artichoke leaf extract (6 weeks of supplementation), plasma cholesterol concentration reduction was associated with a 53% increase in fecal bile acid concentration (66). In Wistar rats fed a HFD for 10 weeks, daily gavage of 40 mg/kg piperine from black pepper increased bile acid excretion in the feces and reduced plasma cholesterol level (67). We cannot explain the difference between our observation and these previous works; however, study design differences (species, intervention, dose, method and duration) may have contributed to these discrepancies. Quantification of the expression of genes encoding enzymes involved in bile acid formation indicated reduced level of *Cyp8b1*and *Cyp27a1* in the WD-T070 groups (Supplementary Figure S1). This suggests that rather than stimulating cholesterol elimination through enhanced bile acid synthesis and fecal excretion, Totum-070 cholesterol-lowering effect is associated with the opposite effect in WD-fed mice. A similar phenotype was described in patients treated with orlistat in whom fecal bile acid amount was decreased (83), while orlistat is known to reduce blood cholesterol level in hypercholesterolemic patients (84). As described earlier, Totum-070 cholesterol-lowering effect might be mediated by inhibition of intestinal cholesterol absorption. Thus, reduced cholesterol elimination though the hepatic bile acid pathway could be compensated by lower cholesterol supply, maintaining Totum-070 cholesterol-lowering global net action.

Recently, the gut microbiota has emerged as a crucial factor that influences cholesterol metabolism. Gut flora modulation by some probiotic and prebiotic agents has cholesterol-lowering property (85). The intestinal microbiota contributes to determine the circulating cholesterol level and may represent a novel therapeutic target in the management of dyslipidemia and cardiovascular diseases (86). Many studies have already linked the microbial community imbalance or maladaptation, defined as “dysbiosis”, to lipid metabolism and metabolic disorders in the host (87, 88). To determine whether Totum-070 hypocholesterolemic effect was associated with the gut microbiota, the association between plasma cholesterol concentration and altered intestinal bacteria at the genus level was tested by Pearson's correlation analysis. The relative abundance of *Muribaculum* was decreased in the WD group but restored by Totum-070 supplementation (Table 3), whereas the relative abondance of *Lactococcus* was increased by WD and reduced by Totum-070 supplementation. Moreover, the relative abundances of *Muribaculum* and of *Lactococcus* were negatively and positively, respectively, correlated with plasma cholesterol. This suggest that *Lactococcus* might have adverse effects on lipid metabolism homeostasis, and *Muribaculum* beneficial effects on plasma cholesterol. This is consistent with previous studies showing that *Muribaculum* relative abundance is lower in WD-fed mice (89) and obese mice fed a HFD (90, 91) than in ND mice, and that *Lactococcus* richness is increased in animals fed a HFD (92, 93). Thus, the present results suggest that *Muribaculum* and *Lactococcus* are bacteria associated with Totum-070 beneficial effect on lipid disorders caused by WD.

It has been reported that members of the *Muribaculum* genus can ferment complex polysaccharides (94), and animal studies demonstrated that increased SCFA production is associated with *Muribaculum* abondance (95, 96). The analysis of the microbiota modifications induced by Totum-070 supplementation highlighted increased abondance of several other genera implicated in SFCA production, such as *Desulfovibrio* (97), *Phocaeicola* (98, 99) and *Parabacteroides* (100–102) for acetate, and *Oscillibacter* (103, 104) and *Ruthenibacterium* (105) for butyrate. Moreover, many *in vitro* studies tested the fermentability of artichoke leaf or olive leaf extracts (included in Totum-070 mix) and demonstrated increased formation of total SCFAs, mainly driven by acetate (106–108). This correlation between increased acetate production upon plant ingredient supplementation and reduction of circulating cholesterol was previously reported. In a clinical trial in patients with hypercholesteremia, oat bran consumption for 3 weeks reduced LDL-C by 12.1% and serum acetate peak was significantly higher than in controls (109). In individuals with normal weight, intake of resistant starch for 4 weeks significantly decreased LDL-C by 5.8% in association with higher acetate concentration (110). In a HFD-fed mouse model, *Astragalus* polysaccharide supplementation improved hepatic steatosis, obesity and dyslipidemia. This effect was dependent on the presence of acetate-producing bacteria (97). Thus, a significant increase in acetate production in humans and animal models contributes to the hypocholesterolemic effects of plant extracts. Although acetate is a lipogenesis substrate in many cells, evidences from animal models suggest that direct administration of acetate improves the lipid profile. In rats fed a cholesterol-rich diet, supplementation with 0.3% acetic acid for 19 days resulted in a significant total cholesterol reduction by 18.7% and a tendency to triglyceride concentration reduction in liver (111). Oral administration of 5.2 mg/kg of acetate for 6 months in obesity-linked type 2 diabetic Otsuka Long-Evans Tokushima Fatty rats protected against fat accumulation in liver and decreased hypercholesterolemia by 35.9% (112). Lastly, meta-analyses of data from clinical trials indicate that dietary acetic acid supplementation significantly reduces triglyceride levels in individuals with overweight and obesity and in patients with type 2 diabetes (113).

In accordance with the effect of Totum-070, consumption of oleanolic acid in mice increased the relative abundance *Oscillibacter* (114). In mice, both tyrosol (115) and hydroxytyrosol (116) increased the relative abundance of family Muribaculaceae. Interestingly, previous report showed that intake of Goji juice in DSS-induced ulcerative colitis mice increased relative abundance of *Oscillibacter*, *Desulfovibrio*, *Parabacteroides* and uncl.*Muribaculaceae* (117), which is consistent with the effects of Totum-070 supplementation. However, the administration of polysaccharides from Goji fruits in streptozotocin-induced mice modified composition of intestinal flora, including increased relative abundance of *Bacteroides* and decreased relative abundance of *Allobaculum* (118), while these last two genera were not significantly regulated by Totum-070. Similar discrepancy was observed with administration of Goji extracts which decreased relative abundance of *Clostridium_XIVa* in HFD-fed mice (26). Contrary to our results, the administration of chlorogenic acid in HFD-fed mice decreased the relative abundance of *Desulfovibrio* and *Bifidobacterium*, while *Bacteroides* was increased (30). These observations demonstrate the complexity behind the various effects generated by the combination of each extract and biomolecule included in Totum-070 mix to produce this specific regulation of intestinal microbiota in WD-fed mice.

Collectively, the present findings indicate that the improved plasma cholesterol level following Totum-070 supplementation, could be, at least partly, mediated by modulation of the gut microbiota and the related acetate production. However, this study has potential limitations. The use of germ-free mice or treatment with broad-spectrum antibiotics to deplete the gut microbiota (119) would give insight in Totum-070 beneficial effects; and also provide knowledge on the importance of the production of SFCAs as a mechanistic part of the Totum-070 hypocholesterolemic effect. Secondly, in order to decipher the molecular mechanism of actions of Totum-070, genetically modified mouse models would be of great interest. For example, mice deficient for the cholesterol transporter NPC1L1 (120) or knock-out for the LDL-receptor (121) would help to understand the cholesterol-lowering effect of Totum-070 in the intestinal cholesterol absorption and through regulation of hepatic LDL-C clearance, respectively. Finally, future good quality clinical studies will have to confirm the hypocholesterolemic potential of this formulation in humans.

In conclusion, we showed that supplementation with Totum-070 prevents the WD-induced plasma cholesterol increase and concomitantly increases fecal lipid excretion and SCFA production. Furthermore, Totum-070 modulated beneficially the gut microbiota composition, by increasing the relative abundance of *Muribaculum* and reducing the relative abundance of *Lactococcus.* These results demonstrates that Totum-070 could be used as an effective dietary supplement to prevent blood cholesterol increase, a major risk factor for cardiovascular disease.

## Data Availability

The datasets presented in this study can be found in online repositories. The 16S rRNA gene sequencing data from mouse fecal microbiota presented in this study has been deposited in the Sequence Read Archive under BioProject PRJNA952522 with the following accession numbers: SRR24071998 to SRR24072026 (BioSamples: SAMN34074972 to SAMN34075000).
