# Supplementary material for: A polyphenol-rich plant extract prevents hypercholesterolemia and modulates gut microbiota in western diet-fed mice
Source: Front Cardiovasc Med. 2024 Jan 22;11:1342388. doi: 10.3389/fcvm.2024.1342388 (PMC10839041; doi:10.3389/fcvm.2024.1342388)
Supplement: Supplementary file 1 [file Datasheet1.docx]

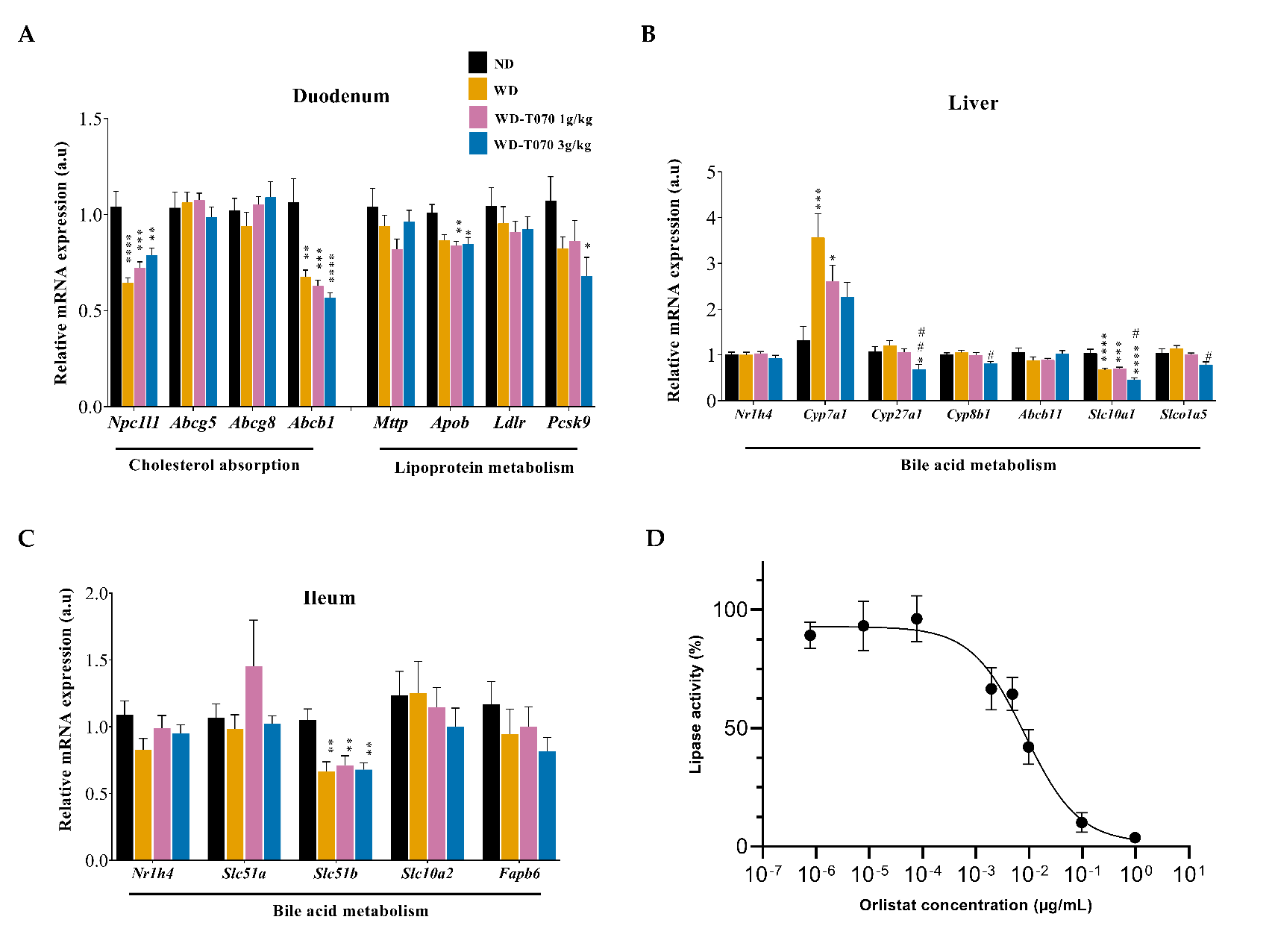
Supplementary Material

**Supplementary Figure S1.** Effect of Totum-070 on duodenum gene expression, bile acid metabolism, and inhibition of pancreatic lipase activity by orlistat (**A**) Relative expression of selected transcripts implicated in cholesterol and lipoprotein metabolism in the duodenum (n=14 per group). (**B**) Relative expression in liver of selected genes encoding proteins implicated in bile acid metabolism (n=14 per group). (**C**) Expression of genes implicated in bile acid homeostasis in the ileum (n=14 per group). (**D**) Inhibition of lipase activity by orlistat, n=4-11 replicates. *p <0.05, **p <0.01, ***p <0.001 and ****p <0.0001 *versus* ND. #p <0.05, ##p < 0.01, ###p <0.001 and ####p <0.0001 Totum-070 groups *versus* WD. Data are the mean ± SEM.
